# Family-Centered Care and Family Well-Being in a Community-Based Adapted Sport Program for Children with Neurodevelopmental Disabilities: A Cross-Sectional Exploratory Study

**DOI:** 10.3390/children13091140

**Published:** 2026-08-26

**Authors:** Francesca Cucinotta, Maria Chiara Scaffidi, Eliana Cipolla, Elvira Maria Mantineo, Giuseppe Santoro, Clara Lombardo, Laura Turriziani, Amerigo Stamile, Sergio Lucio Vinci, Angelo Alito

**Affiliations:** 1 IRCCS Centro Neurolesi Bonino Pulejo, 98124 Messina, Italy; francesca.cucinotta@irccsme.it (F.C.); laura.turriziani@irccsme.it (L.T.); 2 Degree Course in Neuro and Psychomotor Therapy of Developmental Age, Department of Human Pathology of Adulthood and Childhood “Gaetano Barresi”, University of Messina, 98125 Messina, Italy; mariachiarascaffidi@gmail.com; 3 Department of Clinical and Experimental Medicine, University of Messina, 98125 Messina, Italy; eliana.cipolla95@virgilio.it (E.C.); elviramantineo@gmail.com (E.M.M.); 4 Department of Biomedical, Dental Sciences and Morphological and Functional Imaging, University of Messina, 98125 Messina, Italy; giuseppe.santoro@unime.it; 5 Department “Scienze della Salute”, University of Catanzaro, 88100 Catanzaro, Italy; 6 Provincial Health Authority of Cosenza, 87100 Cosenza, Italy; amerigo.stamile@asspcs.it; 7 Brain Mapping Lab, Department of Biomedical, Dental Sciences and Morphological and Functional Imaging, University of Messina, 98125 Messina, Italy; sergio.vinci@unime.it (S.L.V.); alitoa@unime.it (A.A.)

**Keywords:** adapted sport, caregivers, family-centered care, neurodevelopmental disabilities, pediatric rehabilitation, participation, quality of life

## Abstract

**Highlights:**

**What are the main findings?**

- A community sports club with no clinical focus was rated as family-centered as clinical rehabilitation services, except for structured information.
- Acceptance is not connection—children fit in and felt safe yet scored lowest of all on peer support.

**What are the implications of the main findings?**

- Adapted sport is a legitimate setting for family-centered care and deserves recognition in service planning.
- Social participation must be explicitly designed for rather than assumed: acceptance in an inclusive setting does not by itself produce peer connection.

**Abstract:**

Background/Objectives: Family-Centered Care (FCC) is an established standard in pediatric rehabilitation, but it has been studied almost entirely inside health services; whether its principles survive the move into community settings is largely unknown. This paper describes how caregivers perceive the family-centeredness of a community-based adapted sport program and characterizes child quality of life and caregiver well-being. Methods: Cross-sectional study within the “Skill-In” program (University of Messina). Nineteen caregivers of children with neurodevelopmental disabilities completed the MPOC-20, the KIDSCREEN-52 proxy version and the CarerQoL, analyzed with Spearman correlations and bootstrap confidence intervals. Results: The overall MPOC-20 mean item score was 5.43/7 (SD 0.83), concealing a wide spread from Respectful and Supportive Care (6.25) to Providing General Information (3.78). “Not applicable” responses (14.2%) clustered in items presupposing a clinical provider. KIDSCREEN-52 raw sums were converted to Rasch-based T-values (norm: mean 50, SD 10). Eight of ten dimensions fell within half a standard deviation of the norm, including Social Acceptance (47.65); two did not, Social Support and Peers (34.58) and Autonomy (39.42), each over one standard deviation below. Caregivers reported favorable well-being (CarerQoL-VAS 7.32/10), though six of nineteen reported no support. Associations between care processes and family outcomes were weak. Conclusions: A sport organization with no clinical mandate delivered the relational core of family-centered care comparably to rehabilitation services, while falling short on structured information. The children’s quality-of-life profile was normal on most dimensions but selectively low on peer support and autonomy. Acceptance, on this evidence, is not connection: social participation must be designed for, not assumed.

## 1. Introduction

Family-Centered Care (FCC) is a widely recognized care paradigm in pediatric health, particularly in services for children with disabilities and their families [1,2]. It is defined as a care philosophy that promotes structured and continuous collaboration between families and professionals, recognizing the family as the constant in the child’s life and as an essential partner in clinical and rehabilitative decision-making [3,4].

A central component is family empowerment, referring to the development of caregivers’ skills, confidence and self-efficacy in daily management, which is associated with improved psychosocial outcomes, satisfaction and perceived support [2,5].

FCC also requires respect for family values and cultural context, demanding flexibility from services, particularly in pediatric rehabilitation where care must be integrated into daily life [6]. It rests on explicit ethical foundations and promotes family autonomy, although balancing the needs of the child and the family can be ethically complex [7]. It is now regarded not only as a care model but also as an ethical and organizational standard, whose implementation requires changes in clinical practice, staff training and service organization [4,8].

Conventional service models, though effective for specific clinical needs, are limited in promoting lasting change for children and families facing environmental, social and relational challenges [9]. This limitation mirrors the logic of the World Health Organization’s International Classification of Functioning, Disability, and Health (ICF), which locates function not in the child’s body alone but in the dynamic interaction between the child, their activities, their participation, and their environment, and which reframes intervention accordingly [10].

The recent literature therefore emphasizes extending interventions beyond clinical settings into real-life contexts, home, school, leisure and community, where participation naturally occurs [11,12], since embedding participation-based approaches in daily environments facilitates the transfer of therapeutic gains to everyday situations and enhances functional adaptation [13]. Within this framework, sport and physical activity constitute relationally intense ecological environments with strong potential to foster active participation. Adapted or structured community-based sport experiences provide opportunities for social interaction, cooperation and contextual learning, supporting not only motor development but also the building of relationships with peers and adults [14,15]. Such settings are particularly relevant for this population: motor coordination difficulties are common in children on the autism spectrum, providing a specific rationale for structured adapted physical activity [16], and modified sport interventions have shown positive effects on leisure participation in children with disabilities [14]. Participation-oriented physical activity may therefore extend the FCC concept, shifting the focus from treatment toward participation and social inclusion, and creating continuity between health interventions and everyday life [17].

Despite this potential, the evidence base for FCC has been built almost exclusively within health and rehabilitation services, and the psychometric instruments used to evaluate it were developed and validated in those same settings [18]. To our knowledge, whether and to what extent its principles transfer to organizations whose mandate is sporting rather than clinical has not been examined. This gap is not only conceptual but practical: families of children with neurodevelopmental disabilities increasingly access physical activity through community sport programs rather than through health services, yet the quality of what they encounter there—and whether it is family-centered—remains largely undocumented. Characterizing it is a necessary first step toward recognizing, and improving, the care that these families receive outside the clinic.

This study examines a community-based adapted sports program from the perspective of the families using it. We describe caregivers’ perceptions of the program’s family-centeredness and identify its strengths and weaknesses; we characterize proxy-reported child health-related quality of life and caregiver well-being; and we explore, without expectation of statistical power, whether care processes track family outcomes. The rationale for relating these measures is the core hypothesis of the FCC model itself, that family-centered processes are associated with better outcomes for children and the family [19], and testing whether this process–outcome link holds in a non-clinical, community setting is the conceptual aim of this exploration.

## 2. Materials and Methods

### 2.1. Study Design and Setting

This retrospective cross-sectional study analyzed data collected within the “Skill-In” program. Data were collected between July and September 2025, during which written informed consent for participation and for the use of the data for research and publication purposes was obtained from all caregivers. The retrospective analysis of these anonymized data was subsequently approved by the Local Ethics Committee of Messina on 14 April 2026 (record no. 4; protocol no. 130-25), which classified the study as a retrospective–prospective observational study. The study was conducted in accordance with the Declaration of Helsinki. Reporting follows the STROBE statement for cross-sectional studies.

### 2.2. The Skill-In Program

The Skill-In program began in 2019 at the University Sports Citadel in Messina, Italy. Following an integrated sports model, it offers personalized, adapted pathways to athletes with and without disabilities. The program aims to promote psychophysical well-being, autonomy and social inclusion, and to build a stable network of coaches and clubs able to accommodate children with different disabilities. Disciplines available included swimming, archery, blind baseball, artistic gymnastics and sport climbing.

Coaches are recruited through public calls and must hold specific qualifications. Sessions use a one-to-one coach-to-athlete ratio to maximize individualization, and rotation among coaches is deliberately promoted to develop the athletes’ relational and adaptive skills and to reduce emotional dependence on a single adult. The approach draws on play-based motor education for the developmental stage, targeting fundamental movement patterns, coordinative abilities, and sensory-perceptual and conditional capacities.

Families are involved through providing logistical and motivational support, as well as through ongoing informal collaboration with the coaching staff. This helps to personalize individual pathways, monitor progress, and maintain continuity between the home environment and the sporting environment. Sessions ran from October 2024 to June 2025, each lasting 50 min, with an average of two sessions per week.

### 2.3. Participants

Caregivers of children and adolescents with neurodevelopmental disabilities who were enrolled in the program were invited to participate through a convenience sampling strategy. Of the 35 caregiver–child dyads invited, 21 responded (a response rate of 60%). Two were excluded before analysis: one child was over 18 years old, and one was aged 25 and had a non-neurodevelopmental diagnosis (schizophrenia). Both were outside the validated KIDSCREEN-52 age range (8–18 years) and the neurodevelopmental focus of the study, leaving 19 dyads for analysis.

### 2.4. Outcome Measures

Measure of Processes of Care (MPOC-20). The MPOC-20 is a validated self-report measure of parents’ perceptions of the extent to which the services they and their child receive are family-centered [20]. It comprises 20 items across five domains: Enabling and Partnership (three items); Providing General Information (five items); Providing Specific Information about the Child (three items); Coordinated and Comprehensive Care (four items); and Respectful and Supportive Care (five items). Items are rated from 1 (“not at all”) to 7 (“to a very great extent”), with an additional “not applicable” option. Higher scores indicate a greater perception of family-centeredness. Domain scores were computed as the mean of the constituent items. In accordance with the scoring instructions, “not applicable” responses were treated as missing and excluded from computation rather than being assigned a numerical value [20].

The official Italian translation of the MPOC-20, obtained from CanChild (McMaster University), was used. The item wording was adapted to the sports context. The referents “the people who work with your child” denote the coaching and technical staff, and “the organization” denotes the sports club.

KIDSCREEN-52, proxy version. The KIDSCREEN-52 assesses health-related quality of life in children and adolescents aged 8–18 years across ten Rasch-scaled dimensions [21]. The proxy version was completed by caregivers. Items were rated using a 5-point Likert scale. The thirteen negatively worded items (seven Moods and Emotions items, three Self-Perception items, and three Bullying items) were reverse-coded, meaning higher scores consistently indicate a better quality of life. The instrument does not yield a total score, so the ten dimensions are reported separately. Following the manual, the general health item was collapsed to three points before summation. The dimension raw sums were then converted to Rasch person parameters and T-values using the official KIDSCREEN-52 parent/proxy-report conversion table. This table standardizes the scores to a mean of 50 and a standard deviation of 10 in the international norm sample. This conversion was verified against the example provided by the instrument developers. Since the reference sample is from the general population rather than from individuals with disabilities, the T-values are interpreted as deviations from population norms rather than as expected values for this clinical group.

CarerQoL. The CarerQoL measures care-related quality of life of informal caregivers [22]. The CarerQoL-7D assesses the caregiving situation across seven dimensions: two positive (fulfillment and support) and five negative (relational problems, mental health problems, difficulty with daily activities, financial problems, and physical health problems). Each dimension is rated on a scale of three levels: none, some, or a lot. The CarerQoL-VAS measures overall well-being on a scale from 0 (completely unhappy) to 10 (completely happy).

### 2.5. Statistical Analysis

An a priori sample size calculation was not performed. The study was exploratory and enrolled all eligible caregiver–child dyads participating in the Skill-In program during the study period; the final sample size was therefore determined by the size of this single community program rather than by a target calculation. The relatively small sample reflects both the limited prevalence of the population (children with neurodevelopmental disabilities enrolled in a structured adapted-sport program in a defined geographic area) and the exploratory, hypothesis-generating aim of the study, which sought to characterize a setting not previously examined rather than to test predefined effects. Cross-sectional, proxy-report designs of this kind are widely used to characterize health-related quality of life in children with disabilities and in other clinical populations [23,24,25].

Due to the small sample size and ordinal nature of the data, nonparametric methods were adopted. Continuous variables are summarized as means with standard deviations or medians with interquartile ranges, and categorical variables are summarized as frequencies and percentages. Associations between MPOC-20 scores and caregiver or child outcomes were examined using Spearman’s rank correlation coefficient, with 95% confidence intervals obtained by bootstrap resampling (5000 replicates). Given the exploratory design and the small sample, correlations are reported as descriptive measures of the strength and direction of association; no significance testing or correction for multiple comparisons was applied, and the analysis is not intended to support statistical inference. Analyses were performed using Jamovi ver. 2.6.44.

## 3. Results

### 3.1. Participants

Nineteen caregivers completed the study questionnaires. Most children were male (16/19, 84.2%), with a mean age of 11.79 years (SD = 3.19; median = 12.0; range = 6–18). The most frequent diagnosis was Autism Spectrum Disorder (12/19, 63.2%), followed by Down syndrome (5/19, 26.3%), with one child each presenting a rare genetic syndrome and ADHD. Just over a third of the children were non-verbal (7/19, 36.8%), and a similar proportion required partial or constant one-on-one support during sessions (11/19, 57.9%). Caregiver-reported autonomy at home, available for 15 children, was full in 5, partial in 8, and low in 2. The median time enrolled in the program, available for 16 children, was 2 years (IQR 2–5; range 2–7). Artistic gymnastics was the most frequent discipline (9/19, 47.4%). Full participant characteristics are reported in Table 1.

### 3.2. Perceived Family-Centeredness (MPOC-20)

On a scale from 1 to 7, the overall MPOC-20 mean item score was 5.43 (SD = 0.83; median = 5.46; IQR = 5.12–5.90; range = 3.83–6.82), indicating that caregivers perceived the program as family-centered to a fairly great extent, although with substantial variation across domains. However, the domain profile was markedly uneven (Table 2). Respectful and Supportive Care received the highest score (6.25, SD 0.71), followed by Coordinated and Comprehensive Care (5.86, SD 0.91), Enabling and Partnership (5.67, SD 1.05), and Providing Specific Information about the Child (5.37, SD 1.59). “Providing General Information” was substantially lower than every other domain (3.78, SD 1.59).

**Figure 1:**
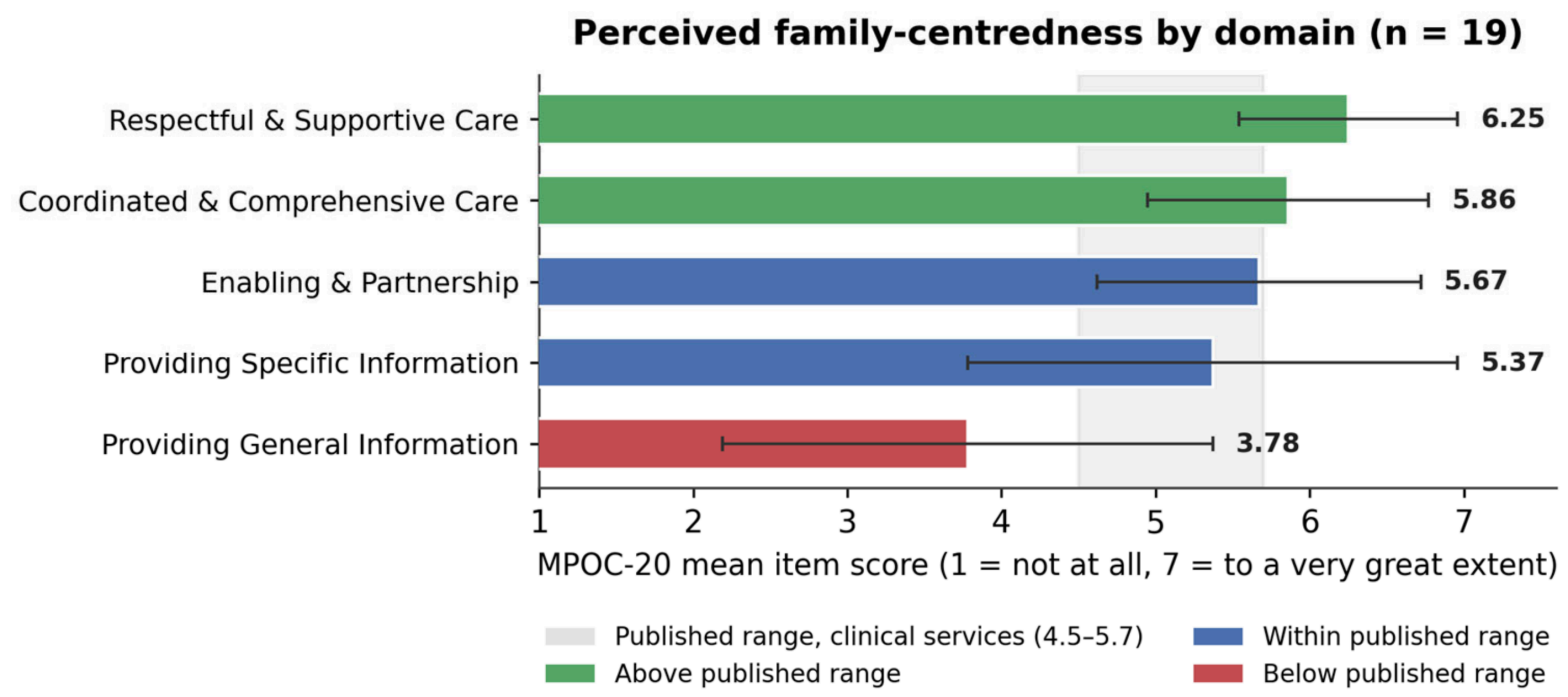
MPOC-20 mean item scores by domain (*n* = 19). Bars show means; error bars, SD. Shaded band, range reported for clinical rehabilitation services (4.5–5.7) [26,27,28,29]. Green, above range; blue, within; red, below.

The domain profile against published values for clinical services is shown in Figure 1.

“Not applicable” responses accounted for 54 of the 380 responses to the items (14.2%), reported by 12 of the 19 caregivers. These responses were not distributed randomly; rather, they clustered around items presupposing a clinical service. These items included written information on the child’s progress (9/19, 47.4%), information materials such as booklets, kits, or videos (9/19, 47.4%), information about the child’s condition, its causes, and prognosis (8/19, 42.1%), opportunities to make decisions about activities (6/19, 31.6%), and continuity of the same reference person over time (5/19, 26.3%). Five items received no “not applicable” responses, all of which belonged to the relational domains.

### 3.3. Child Health-Related Quality of Life (KIDSCREEN-52 Proxy)

Table 3 shows the raw dimension sums and their corresponding T-values. The profile was selective rather than uniformly depressed.

Overall, the proxy-reported quality-of-life profile was close to the general population norm on most dimensions. Eight out of ten fell within half a standard deviation of the reference mean of 50, while two dimensions (i.e., Autonomy and Social Support and Peers) fell more than one standard deviation below it.

These dimensions were Self-Perception (48.72), Financial Resources (48.47), Parent Relations and Home Life (47.96), Social Acceptance (47.65), School Environment (47.46), Moods and Emotions (46.07), Physical Well-Being (45.86), and Psychological Well-Being (45.59).

**Figure 2:**
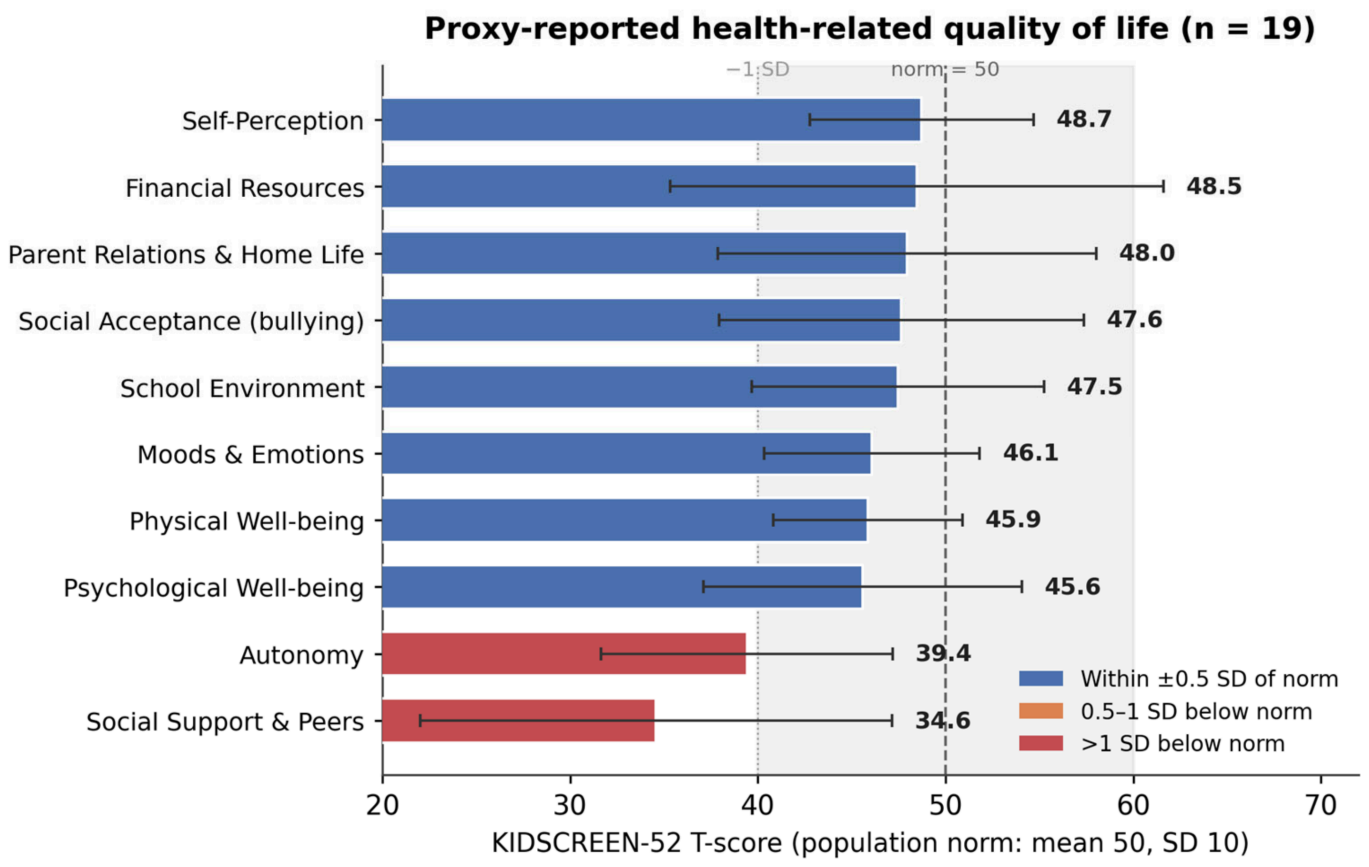
Proxy-reported KIDSCREEN-52 dimension T-values (*n* = 19; norm: mean 50, SD 10), ordered highest to lowest. Bars show means; error bars, SD. Dashed line, norm (T = 50); shaded band, ±1 SD; dotted line, T = 40. Blue, within ±0.5 SD; red, >1 SD below. Higher = better quality of life.

Two dimensions stood apart. Social Support and Peers reached a mean T-value of 34.58 (SD 12.57), which is more than one and a half standard deviations below the norm. Twelve of the nineteen children scored below T = 40. Autonomy reached 39.42 (SD 7.77), with 11 of the 19 children scoring below T = 40. Both dimensions also showed the greatest dispersion of the profile, indicating that the deficit was not uniform across the sample. These T-values are referenced to a general-population (neurotypical) norm; in a cohort composed predominantly of children with autism spectrum disorder and Down syndrome, some reduction relative to that baseline is expected. The interpretation of this profile is considered in the Discussion. The full profile, with each dimension referenced to the population norm, is shown in Figure 2.

### 3.4. Caregiver Outcomes (CarerQoL)

On the CarerQoL-VAS (range 0–10), caregiver well-being was favorable overall, with a mean of 7.32 (SD 1.42; median 8.00), indicating that most caregivers rated their overall situation positively, as no Italian tariff is available for the CarerQoL-7D.

The CarerQoL-7D dimensions are categorical (none/some/a lot) and are therefore reported as response frequencies; a weighted utility score was not computed. The dimension profile (Table 4) showed a high sense of fulfillment, which was reported as substantial by 14/19 caregivers and as absent by none. Relational and financial problems were largely absent, with 14 and 13 caregivers, respectively, reporting none. Conversely, nine caregivers reported some mental health problems, twelve reported some or considerable difficulty combining caregiving with daily activities, and eight reported some or considerable physical health problems. Perceived support was the most heterogeneous dimension: seven caregivers reported substantial support, while six reported none.

### 3.5. Associations Between Care Processes and Family Outcomes

Associations between perceived family-centeredness and caregiver or child outcomes were uniformly weak and imprecise (Table 5). All coefficients were small in magnitude, and every bootstrap confidence interval spanned zero, indicating associations compatible with effects in either direction. The overall MPOC-20 score was essentially unrelated to caregiver well-being (ρ = 0.058, 95% CI −0.36 to 0.49) and to proxy-reported child quality of life across dimensions (all |ρ| ≤ 0.19). The strongest single association observed was an inverse relationship between Enabling and Partnership and Physical Well-being (ρ = −0.537, 95% CI −0.75 to −0.20); given the exploratory nature of the analysis and the number of coefficients examined, this is reported descriptively and is not interpreted as evidence of an effect. Consistent with the study’s design, these results are presented to characterize the pattern of associations in the sample, not to test hypotheses.

## 4. Discussion

Caregivers rated the Skill-In program as fairly family-centered. However, the overall score of 5.43/7 conceals an uneven profile. The relational domains scored highly, but the provision of general information did not. The children’s quality-of-life profile was different, falling close to population norms on eight dimensions but more than one standard deviation below on peer support and autonomy. Caregiver well-being was favorable, though not uniform. Associations between care processes and family outcomes were weak.

The relational scores are worth considering. Across international MPOC-20 studies, domain means range from approximately 4.5 to 5.7, and the ordering is remarkably consistent. Some form of respectful, coordinated care is at the top, and the provision of general information is at the bottom [26,27,28,29]. Our sample reproduces that ordering but sits higher on the relational side. Respectful and Supportive Care is at 6.25, and Coordinated and Comprehensive Care is at 5.86. In other words, a sports club with no clinical mandate delivered the relational core of family-centered care at least as well as the health services against which the instrument was originally calibrated.

There are structural reasons why this might be so. Coaches work one-on-one with athletes, families are present at every session, and contact between staff and parents is continuous and informal. The frequency of contact, the ordinary setting, and the absence of the asymmetry built into a clinical encounter all plausibly favor what the relational items ask about: being treated as a person rather than as a patient’s parent, being given time without feeling rushed, and meeting genuine interest in the child as a whole. None of this requires a therapeutic mandate. It requires proximity and continuity, which a sports club can provide more easily than an outpatient service.

Information is the mirror image. General Information scored 3.78, below the top domain. This is unsurprising as the respondent is rating SSD UniMe, a sport club without the competence to explain a child’s condition.

However, the way a club communicates is within its control, rather than the clinical content of its communication. The framing and delivery of information shapes how families experience it and how much they trust providers [2,30,31]. This relational dimension of information-giving is well within a sports organization’s reach.

The pattern of missing responses corroborates that reading, which we consider a finding in its own right. Fourteen percent of the MPOC-20 responses were marked “not applicable,” and they were not distributed at random. They concentrated on items regarding written progress reports, informational booklets and videos, information about the disability itself, and the continuity of a single case-holding professional. Not a single caregiver marked a relational item as not applicable. Thus, a substantial part of the instrument simply does not describe what happens in a gym. This has practical implications for anyone analyzing such data. If “not applicable” is scored as a low value rather than treated as missing—as can easily happen when responses are exported from an online form—the result is a systematic penalty against non-clinical providers. An artifact of instrument fit is mistaken for a deficiency of the service. In our dataset, the difference between the two approaches was 0.8 points on a seven-point scale.

Future sport-based research could prevent this artifact in two ways: as an analytic rule, “not applicable” responses should be treated as missing rather than recoded as low values; and, more substantially, a context-specific short form could retain the relational domains—which transferred well here—while reformulating the information items that presuppose a clinical mandate.

The quality-of-life findings are uncomfortable in one respect and are worth stating in normative terms because raw dimension scores are not comparable with one another. On eight of the ten dimensions, these children were within half a standard deviation of the general population norm, including social acceptance at T = 47.65. This means they were mocked, feared, or bullied no more than their peers in the community at large. Two dimensions departed sharply from that picture: Social Support and Peers, with a score of 34.58, and Autonomy, with a score of 39.42. Each score was more than one standard deviation below the norm, with about six out of ten children falling below T = 40 on each dimension.

The shape of this profile is more important than any single value. Instead of the general effect of disability on quality of life depressing all ten dimensions, we observed a normal profile with two deep and specific troughs in the domains of reciprocal peer relationships and independence. Emotional well-being, self-perception, family relationships, and school experience were unremarkable. According to their caregivers, these children were accepted but largely without friends.

The composition of the sample lends this pattern a plausible interpretation, though not a conclusive one. Autism spectrum disorder was the most frequent diagnosis (12/19), and difficulties in reciprocal social interaction are a defining feature of the condition rather than an incidental one. A selective trough in peer support and, to a lesser degree, autonomy is coherent with that. We report this as an observation about internal consistency, not as a stratified finding. With nineteen children and four diagnostic groups of very unequal size, formal comparison across diagnoses would be underpowered and potentially misleading. We did not attempt it.

This reading gains coherence from the two diagnoses that dominate the sample. In autism, reduced peer support is central to the condition [32,33,34]. Even in mainstream settings, autistic children tend to occupy fewer central positions in peer networks and form fewer and less reciprocal friendships, which maps directly onto the low Social Support and Peers score. Down syndrome, the second most frequent diagnosis, shows a complementary profile: socialization is a relative strength, but everyday autonomy is constrained by executive function difficulties [35,36]. Thus, the peer-support trough is plausibly driven by the autistic majority, while the low autonomy is driven by the Down syndrome profile. This interpretive alignment with sample composition makes a purely artifactual explanation less likely. Beyond confirming the expected diagnosis, this alignment has its own interpretive implications: the program did not create or correct the peer-support deficit—it left a pre-existing, diagnosis-linked vulnerability untouched. This is precisely the area in which a redesigned, peer-oriented format could provide added value that the current structure lacks.

It is appealing to interpret the low peer support score as a trait of the children rather than the setting. With this type of sample, that cannot be ruled out. However, the selectivity of the deficit argues against a purely dispositional interpretation since the same children were unremarkable on eight other dimensions. One feature of the program deserves examination. The one-to-one coach-to-athlete ratio, which was adopted to maximize individualization and safety, organizes the session around a child-adult dyad. Coach rotation, introduced to prevent excessive attachment to one adult, operates within that dyad rather than outside it. Neither arrangement provides opportunities for children to interact with each other. If social participation is an objective of an inclusive program and not merely a hoped-for byproduct, then a format that keeps children safe and accepted while leaving them without peers is a legitimate target for revision. This could include paired or small-group segments inside the session, peer-mediated tasks, and cooperative rather than parallel activities. The program could also accommodate mixed groups of athletes with and without disabilities, as the integrated-sport model it already espouses would allow. This is a hypothesis, and a cross-sectional study cannot test it. However, it is testable and comes from the data rather than from first principles.

The literature on adapted sports helps explain why a welcoming environment alone does not necessarily generate friendships. Sports intervention programs for children with intellectual disabilities and autism have been shown to consistently improve physical health, emotional well-being, and a general sense of social inclusion [37]. Physical activity has also been shown to support the development of sports-based friendships in autistic adolescents [38]. However, the social benefits are not automatic. Reviews of these programs indicate that gains in reciprocal relationships depend on the structure of the activity—specifically, collaborative, group-based, and unified formats that deliberately engineer peer interaction—rather than on participation itself. This is precisely the distinction that our data reveal. A one-on-one format provides acceptance, safety, and individual progression. However, it does not provide the peer interaction necessary for forming friendships. The solution is not more sports, but sports with a different structure. When considered alongside our data, this literature suggests a specific conclusion: in this programme, the amount of sport was adequate, but it was not designed to encourage peer interaction. The relational quality that families valued was delivered through the very one-to-one structure that limited horizontal contact among the children. This is our central interpretive claim, which reframes the peer-support deficit as a by-product of a design choice that can be revised, rather than a failure of the program.

Caregiver well-being was favorable (VAS 7.32), and the sense of fulfillment in the caregiving role was high. Notably, not a single caregiver reported the absence of this sense of fulfillment, which is consistent with the relational strengths described above and with evidence that family-oriented services contribute to parental wellness [5]. The rest of the picture is less rosy. About half of the caregivers reported mental health issues and difficulty balancing care with daily activities. Six of the nineteen caregivers reported receiving no support. The quality of life related to care is shaped by clinical, economic, and organizational determinants that are beyond the scope of a sports program. Therefore, a good relational environment should not be expected to neutralize these factors.

Associations between perceived family-centeredness and family outcomes were weak throughout, with confidence intervals wide enough to accommodate effects in either direction. With only nineteen participants, large associations were the only ones that could be detected, so these results are uninformative rather than negative. Nevertheless, two remarks are worth making. First, conceptually, generic health-related quality of life is multiply determined, and process-of-care measures have been reported to indirectly influence family outcomes through satisfaction and perceived support rather than through generic well-being indices [19].

Methodologically, the strongest association we observed—an inverse relationship between Enabling and Partnership and Physical Well-being—was still weak and imprecise, and we treat it as descriptive only. Its direction is nonetheless consistent with a need-driven pattern, in which families of children with greater functional limitation experience more intensive partnership; separating such a pattern from noise would require a larger sample and adjustment for the child’s functional level, which our data did not permit.

What follows for practice? Adapted sports should be recognized as settings for family-centered care, and their relational quality should be acknowledged when planning or funding such programs. Closing the informational gap is the most tractable target, and it does not require the club to acquire clinical competence. Rather, it requires a working channel to the services that already follow these children so that clinical information reaches families by the appropriate route. Meanwhile, the club should take responsibility for providing clear and consistent information about its own activities, aims, and the child’s progress within them. The peer-support finding points elsewhere. It suggests that social participation must be designed with its own methods and indicators rather than assuming it will follow from simply putting children in the same room. Acceptance and connection are not the same outcome, and only one appears to be achieved here.

### 4.1. Limitations

The study has several limitations. The sample was small, drawn from a single program, and mostly male, which limits the inferences about girls and their families. The cross-sectional design precludes any statement about causality or changes over time. Without a comparison group, the observed profile cannot be separated from the characteristics of families who self-select into the program. Diagnosis, communication level, and in-session support were available, but the sample was too small and distributed too unevenly to support stratified or adjusted analysis. The process–outcome correlations are therefore unadjusted for clinical profile. The analysis was retrospective and the questionnaire was anonymous, a deliberate choice given that the data concern minors with disabilities. This meant that caregiver characteristics were not captured and could not be reconstructed. All child outcomes were proxy-reported, which is a real constraint when the construct at issue is the child’s own experience of friendship. The MPOC-20 has not been formally validated in Italian, and the adaptation of its referents to a sport setting was not psychometrically re-validated; both may affect the comparability of scores. Finally, the KIDSCREEN norms derive from a general-population European sample rather than from children with neurodevelopmental disabilities. The deviations therefore describe distance from the general population rather than from appropriate clinical reference.

### 4.2. Future Directions

Longitudinal designs with larger, multi-center samples are the obvious next step. They would allow adjustment for diagnosis and functional level, as well as for whether sustained participation changes anything. A comparison between families in conventional clinical rehabilitation and families in community-based adapted sports would clarify how the two settings complement each other. This comparison is foreseen within the approved protocol and is the direction we intend to pursue. Child-reported outcomes should be incorporated wherever the child’s communicative profile allows, using accessible or adapted formats. This matters most for the social dimensions, which are precisely where proxy report is weakest. The hypothesis about session format is directly testable. Compare sessions with structured peer-mediated components against the current one-to-one arrangement and see whether peer support moves.

## 5. Conclusions

Caregivers of children with neurodevelopmental disabilities found this adapted sports program to be respectful, supportive, and collaborative. It compares favorably with published figures from clinical pediatric rehabilitation. However, caregivers found it lacking in structured information, which reflects what a sports club is and is not equipped to provide. The children were accepted and emotionally at ease. On eight of ten quality-of-life dimensions, including bullying, they were indistinguishable from general population norms. However, on peer support and autonomy, they were more than one standard deviation below. Inclusion, based on this evidence, protects before it connects. Whether a change in session format would close that gap is a question that the present design cannot answer, but it can pose the question precisely.

## Figures and Tables

**Table 1:** Participant characteristics (*n* = 19).

Characteristic	Value
Child sex, male/female	16 (84.2%)/3 (15.8%)
Child age, mean (SD), years	11.79 (3.19)
Child age, median (range), years	12.0 (6–18)
Diagnosis—Autism Spectrum Disorder	12 (63.2%)
Diagnosis—Down Syndrome	5 (26.3%)
Diagnosis—Rare Genetic Syndrome	1 (5.3%)
Diagnosis—ADHD	1 (5.3%)
Communication—verbal	7 (36.8%)
Communication—verbal, simple sentences	5 (26.3%)
Communication—non-verbal	7 (36.8%)
In-session support—independent	8 (42.1%)
In-session support—partial	8 (42.1%)
In-session support—constant	3 (15.8%)
Home autonomy (*n* = 15) full/partial/low	5/8/2
Years in program (*n* = 16), median	2 (2–5)
Discipline—artistic gymnastics	9 (47.4%)
Discipline—gymnastics	4 (21.1%)
Discipline—swimming	2 (10.5%)
Discipline—combined activities	4 (21.1%)

The percentages are based on the analysis of 19 dyads. Home autonomy and time spent on the program were not recorded for all participants; the denominators are stated.

**Table 2:** MPOC-20 domain scores (*n* = 19).

Domain	Items	Mean (SD)	Median [IQR]	Range
Respectful and Supportive Care	5	6.25 (0.71)	6.20 [6.00–6.80]	4.20–7.00
Coordinated and Comprehensive Care	4	5.86 (0.91)	6.00 [5.38–6.71]	4.00–7.00
Enabling and Partnership	3	5.67 (1.05)	6.00 [5.42–6.33]	3.33–7.00
Providing Specific Information	3	5.37 (1.59)	6.00 [4.83–6.50]	2.00–7.00
Providing General Information	5	3.78 (1.59)	4.00 [2.00–4.75]	1.40–6.67
Overall mean item score	20	5.43 (0.83)	5.46 [5.12–5.90]	3.83–6.82

Scores range from 1 (not at all) to 7 (to a very great extent). In accordance with the scoring instructions, “not applicable” responses were excluded from the computation.

**Table 3:** KIDSCREEN-52 proxy dimension scores (*n* = 19).

Dimension	Raw Sum, Mean (SD)	T-Value, Mean (SD)	T-Value, Median [Range]	*n* with T < 40
Self-Perception	20.5 (2.4)	48.72 (5.96)	49.1 [35.8–61.4]	1
Financial Resources	10.6 (4.1)	48.47 (13.13)	51.9 [24.0–65.0]	4
Parent Relations and Home Life	23.9 (4.0)	47.96 (10.08)	46.9 [25.5–69.2]	4
Social Acceptance (bullying)	13.3 (1.7)	47.65 (9.73)	44.8 [27.6–58.8]	5
School Environment	21.7 (3.7)	47.46 (7.77)	49.8 [30.9–62.5]	5
Moods and Emotions	28.7 (2.6)	46.07 (5.75)	46.1 [34.6–54.4]	3
Physical Well-being	16.7 (1.7)	45.86 (5.05)	43.7 [38.8–59.4]	2
Psychological Well-being	22.5 (3.3)	45.59 (8.48)	46.0 [29.2–61.1]	7
Autonomy	16.0 (3.3)	39.42 (7.77)	39.5 [15.7–53.9]	11
Social Support and Peers	15.5 (5.2)	34.58 (12.57)	36.7 [8.3–55.4]	12

T-values are standardized to a mean of 50 and a standard deviation of 10 in the international KIDSCREEN normative sample (general population, parent/proxy report). Higher values indicate a better quality of life. The KIDSCREEN-52 does not provide a total score.

**Table 4:** CarerQoL-7D dimension frequencies (*n* = 19).

Dimension	None	Some	A Lot
Fulfillment from caregiving (positive)	0	5	14
Support in caregiving (positive)	6	6	7
Relational problems	14	5	0
Mental health problems	10	9	0
Problems combining daily activities	7	10	2
Financial problems	13	6	0
Physical health problems	11	7	1

For the two positive dimensions, “a lot” indicates a favorable situation. For the five negative dimensions, “none” indicates a favorable situation.

**Table 5:** Spearman correlations between MPOC-20 scores and family outcomes (*n* = 19).

Pair	ρ	95% CI
MPOC-20 overall × CarerQoL-VAS	0.058	−0.36 to 0.49
MPOC-20 overall × Physical Well-being	−0.187	−0.59 to 0.26
MPOC-20 overall × Psychological Well-being	0.154	−0.35 to 0.59
MPOC-20 overall × Social Support and Peers	0.064	−0.44 to 0.58
MPOC-20 overall × Autonomy	0.136	−0.45 to 0.67
Enabling and Partnership × Physical Well-being	−0.537	−0.75 to −0.20

Spearman rank correlations with 95% confidence intervals (bootstrap, 5000 replicates). Coefficients are reported descriptively; no significance testing was performed.

## Data Availability

The data supporting the findings of this study are not publicly available due to privacy and ethical restrictions concerning a vulnerable population but may be available from the corresponding author upon reasonable request.
